# Predictors of one-year outcomes in chronic heart failure: the portrait of a middle income country

**DOI:** 10.1186/s12872-019-1226-9

**Published:** 2019-11-09

**Authors:** Luciana Gioli-Pereira, Fabiana G. Marcondes-Braga, Sabrina Bernardez-Pereira, Fernando Bacal, Fábio Fernandes, Alfredo J. Mansur, Alexandre C. Pereira, José E. Krieger

**Affiliations:** 1 Laboratory of Genetics and Molecular Cardiology, Heart Institute (InCor) of University of São Paulo Medical School, Avenue Dr. Enéas de Carvalho, Aguiar, 44 Cerqueira César, São Paulo, SP 05403-000 Brazil; 2 Heart Transplant Department, Heart Institute (InCor) of University of São Paulo Medical School, São Paulo, Brazil; 3 Heart Institute (InCor) of University of São Paulo Medical School, São Paulo, Brazil

**Keywords:** Systolic heart failure, Outcomes, Mortality predictors

## Abstract

**Background:**

Heart failure (HF) is a major public health problem with increasing prevalence worldwide. It is associated with high mortality and poor quality of life due to recurrent and costly hospital admissions. Several studies have been conducted to describe HF risk predictors in different races, countries and health systems. Nonetheless, understanding population-specific determinants of HF outcomes remains a great challenge.

We aim to evaluate predictors of 1-year survival of individuals with systolic heart failure from the GENIUS-HF cohort.

**Methods:**

We enrolled 700 consecutive patients with systolic heart failure from the SPA outpatient clinic of the Heart Institute, a tertiary health-center in Sao Paulo, Brazil. Inclusion criteria were age between 18 and 80 years old with heart failure diagnosis of different etiologies and left ventricular ejection fraction ≤50% in the previous 2 years of enrollment on the cohort. We recorded baseline demographic and clinical characteristics and followed-up patients at 6 months intervals by telephone interview. Study data were collected and data quality assurance by the Research Electronic Data Capture tools. Time to death was studied using Cox proportional hazards models adjusted for demographic, clinical and socioeconomic variables and medication use.

**Results:**

We screened 2314 consecutive patients for eligibility and enrolled 700 participants.

The overall mortality was 6.8% (47 patients); the composite outcome of death and hospitalization was 17.7% (123 patients) and 1% (7 patients) have been submitted to heart transplantation after one year of enrollment. After multivariate adjustment, baseline values of blood urea nitrogen (HR 1.017; CI 95% 1.008–1.027; *p* < 0.001), brain natriuretic peptide (HR 1.695; CI 95% 1.347–2.134; p < 0.001) and systolic blood pressure (HR 0.982;CI 95% 0.969–0.995; *p* = 0.008) were independently associated with death within 1 year. Kaplan Meier curves showed that ischemic patients have worse survival free of death and hospitalization compared to other etiologies.

**Conclusions:**

High levels of BUN and BNP and low systolic blood pressure were independent predictors of one-year overall mortality in our sample.

**Trial registration:**

Current Controlled Trials NTC02043431, retrospectively registered at in January 23, 2014.

## Background

Heart failure (HF) is a major public health problem with increasing prevalence worldwide [1]. Once established, worsening heart failure is frequent and associated with significantly diminished quality of life, recurrent hospital admissions and direct impacts in healthcare costs [2]. The estimated prevalence of HF is 1 to 2% of the adult population in developed countries [3]. In Brazil, the HF prevalence is 2 million patients and its incidence is 240,000 new cases per year [4].

Despite the fact that most data on outcomes in patients with HF come from North America and Europe [5]; recently, several studies reported the risk factor prevalence and mortality predictors variation among races [6–8]. Brazil has the largest universal health system in the world; in addition, it is characterized by intense racial admixture, social inequalities and cultural traditions that may impact the natural history of HF. Finally, comprehensive epidemiological, clinical and therapeutic data on chronic HF are still lacking and making the definition of population strategies for disease treatment and prevention at the least difficult to forecast.

In this scenario, we have conducted the GENIUS-HF (Genetic and ElectroNic medIcal records to predict oUtcomeS in Heart Failure patients) study, a Brazilian cohort that aims to contribute with the characterization of risk predictors and the impact of multimorbidity related to chronic HF [9].

The purpose of this study was to describe baseline characteristics, one-year outcomes and predictors of mortality and hospitalization of chronic heart failure patients.

## Methods

### The cohort

**Fig. 1:**
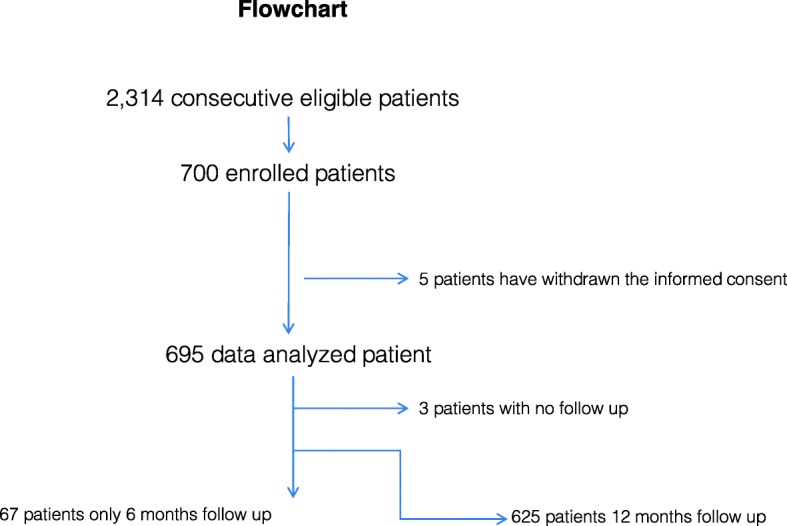
Flowchart. Reasons for patients exclusion were: Ejection fraction > = 50% or echocardiogram with a date exceeding 2 years of acquisition (316 patients); Patients without echocardiogram at the time of the invitation - clinical diagnosis of HF only (587 patients); Age > 80 years at the time of inclusion (84 patients); Patient without telephone contact for recruitment (334 patients); Refusal of the eligible patient or responsible person to participate (230 patients); Death before the invitation for inclusion (63 patients)

GENIUS-HF (Genetic and ElectroNic medIcal records to predict oUtcomeS in Heart Failure patients) is an observational, prospective, single-center cohort started in 2012 and still ongoing. Invited individuals were patients with chronic systolic heart failure sequentially seen at the SPA outpatient clinic at the Heart Institute, University of São Paulo Medical School (InCor - HCFMUSP). The SPA clinic is an outpatient clinic responsible for triaging patients from primary and secondary care sites to a tertiary care site. The rationale and design for this study have been previously published [9]. Since 2012, 700 patients were included from 2314 individuals screened at the outpatient clinic due to HF symptoms (Fig. 1: Flowchart). Enrolled patients were submitted to a clinical baseline evaluation, complementary exams (echocardiography, cardiograph impedance) and biochemical tests, which included blood, urine and biobanking samples for future analysis. After enrollment, patients would be taken care at different units of the public medical system and study investigators did not influence any of the medical decisions involving participants. Follow-up was made thru phone interview every six-months.

### Ethical aspects

The study protocol was approved by the Ethics Committee for Medical Research on Human Beings of the Clinical Hospital from University of São Paulo Medical School (Protocol number SDC 2368/03/162). Signed informed consent was obtained from all participants. This study was retrospectively registered at clinicaltrials.gov (NTC02043431) in January 23, 2014.

### Eligibility criteria

Patients between 18 and 80 years old and with systolic heart failure diagnosis from different etiologies were eligible for enrollment in the cohort. The left ventricular ejection fraction ≤50% was confirmed on two-dimensional transthoracic Doppler echocardiography performed in the past 2 years before enrollment. The diagnosis of heart failure was made according to previously published criteria [10]. To define the appropriate etiology of cardiomyopathy, the authors followed previous guidelines [11, 12]. Patients with impaired cognition due to advanced dementia or severe psychiatric disorder, without telephone access or that refused to participate in follow-up procedures were not eligible.

### Studied variables

Data collection included demographic variables (sex, ethnicity, age) duration of symptoms, etiology of heart failure, comorbidities, smoking status, body mass index, heart rate, blood pressure, cardiac rhythm, and cardiac dimensions, left ventricle ejection fraction, medication use and clinical outcomes.

### Outcomes

Patients included have been submitted to telephone follow-up in 6 and 12-months to assess cardiovascular outcomes: all-cause mortality, hospitalization and heart transplantation. Regarding the 67 patients that had only the first telephone contact, we consider the events occurred in this period of follow up. All events were adjudicated by study investigators.

### Statistical analysis

Means and SD were calculated for continuous variables, counts and percentages for categorical.

variables, and median (IQR) for BNP due to non-normality. We performed logarithmic.

transformation of BNP in order to normalize the sample data for analysis. Baseline characteristics were compared using One-way ANOVA followed by Dunnet for continuous variables and Chi-square test for categorical variables. The prognostic value was tested by univariate and multivariate Cox proportional hazard analysis. The multivariate analysis was constructed with the significant variables in the bivariate model. A value of *p* < 0.05 was considered statistically significant for all comparisons. These analyses were performed using a statistical software package e (SPSS ver. 20.0, IBM, Armonk, NY, USA).

### Results

Since 2012 we screened 2314 consecutive patients for eligibility and enrolled 700 participants. This paper describes the prognosis value of clinical baseline and laboratorial characteristics of the sample.

Five individuals have withdrawn the informed consent and were excluded from the study. Among 695 participants, 3 (0.4%) individuals did not respond to any follow-up contact and 67 (9.6%) have only the 6-month contact (Fig. 1, flowchart).

The overall one-year mortality was 6.8% (47 patients) and the composite outcome of death or hospitalization was 17.7% (123 patients). Seven patients (1%) have been submitted to heart transplantation during one year of enrollment.

The baseline characteristics of the individuals (Table 1) showed a mean age of 55.4 years old and male gender predominance (67.6%) as well as self-referred mixed race (49.6%). The main comorbidities were dyslipidemias (66.5%) followed by hypertension (64.5%), diabetes (29.5%) and chronic kidney disease (26.9%). In this sample, 9.2% were previous or current smokers.

**Table 1:** Baseline demographic and clinical characteristics

Variable	Total (*n* = 695)
Age (years)	55.4 + 12.2
Gender (Male) n(%)	470 (67.6)
*Race n(%)*	
Asian	7 (1.0)
Black	107 (15.4)
Mixed	345 (49.6)
White	236 (34.0)
Hypertension n(%)	448 (64.5)
Diabetes n(%)	205 (29.5)
Dyslipidemias n(%)	462 (66.5)
Smoking n(%)	64 (9.2)
Chronic Renal Failure n(%)	187 (26.9)
COPD n(%)	49 (0.07)
CABG n(%)	34 (0.04)
PCI n(%)	50 (0.07)
HIV n(%)	2 (0.003)
*Heart Failure Class n(%)*	
NYHA I	130 (18.7)
NYHA II	433 (62.3)
NYHA III	127 (18.3)
NYHA IV	5 (0.7)
Ejection Fraction (%)	32.0 + 8.6
LVDD n(%)	64.1 + 8.3
*Heart failure etiology n(%)*	
hypertensive	181 (26.0)
ischemic	152 (21.9)
chagasic	118 (17.0)
idiopathic	108 (19.6)
other	136 (8.6)
Weight (Kg)	75.8 + 19.1
Body mass index (kg/m2)	27.9 + 6.0
Heart rate (bpm)	71.2 + 14.3
Systolic blood pressure (mmHg)	123.5 + 23.8
Diastolic blood pressure (mmHg)	76.2 + 14.6
Dyspnea n(%)	599 (86.2)
Orthopnea n(%)	263 (37.8)
Paroxysmal nocturnal dyspnea n(%)	150 (21,6)
Jugular venous distension n(%)	250 (36.0)
Pulmonary rales n(%)	52 (7.5)
Peripheral edema n(%)	127 (18.3)
Third heart sound n(%)	50 (7.2)
Hepatojugular reflux n(%)	71 (10.2)
Capillary filling time (3–5 s) n(%)	19 (2.7)
Ascitis n(%)	18 (2.6)
Hepatomegaly n(%)	79 (11.4)
Creatinine (mg/dL)	1.27 + 0.77
Blood urea nitrogen (mg/dL)	49.3 + 23.7
CKD-EPI (μmol/L)	68.2 + 22.7
Sodium (mg/dL)	139.3 + 2.72
Potassium (mg/dL)	4.8 + 0.6
Hemoglobin (mg/dL)	13.9 + 1.7
Hematocrit (%)	43.0 + 5.2
Blood glucose fasting (mg/dL)	113.6 + 51.2
Glycated hemoglobin (%)	6.3 + 0.7
High sensitive troponin (ng/dL)	0.040 + 0.062
BNP (pg/mL)	149 (54–355)
*Medication in use n (%)*	
Beta blocker	673 (96.8)
ACE inhibitor	427 (61.4)
ARB	207 (30.0)
Nitrate	98 (14.1)
Hydralazine	116 (16.7)
Diuretic	631 (90.8)
Digital	165 (23.7)
Lipid lowering	332 (47.8)

Continuous variables are expressed as mean ± SD

Categorical variables are presented as absolute number and percentage [n (%)]

BNP was expressed as median (interquartile range) due to non-normality

*COPD* Chronic Obstructive Pulmonary Disease, *CABG* Coronary Artery Bypass Grafting, *PCI* Percutaneous Coronary Intervention, *HIV* Human Immunodeficiency Virus, *NYHA* New York Heart Association, *LVDD* left ventricular end diastolic dimension, *CKD-EPI* Chronic Disease Epidemiology Collaboration, *BNP* brain natriuretic peptide, *ACEi* angiotensin converting enzyme inhibitor, *ARB* angiotensin II receptor blocker

Regarding heart failure etiology distribution, we observed a predominance of hypertensive (26.0%), ischemic (21.9%) and chagasic (17.0%) forms of cardiomyopathy. Most of the included individuals were in NYHA class I/II (81%) at enrollment.

Mean BMI (body mass index) was 27.9 kg/m2. Eighty six percent of the patients reported dyspnea as a symptom and jugular venous distension was the most observed clinical sign at examination (36% of the patients). Median BNP (brain natriuretic peptide) was 149 pg/mL (interquartile range: 54–355). Regarding medication, 96.8% of patients were in use of a beta-blocker; 91.1% used ACEi or ARB medication and 90.8% of the patients were in use of some diuretic at baseline.

Table 2 presents results for the Cox proportional hazards regression model estimated in the cohort using all pre-specified clinical and demographic characteristics. After multivariate adjustment, BUN (hazard ratio [HR] 1.017; 95% CI 1.008–1.027), Log BNP (hazard ratio [HR] 1.695; 95% CI 1.347–2.1134) and systolic blood pressure (hazard ratio [HR] 0.982; 95% CI 0.969–0.995) were independently associated with death within 1 year.

**Table 2:** Variables associated with all-cause mortality at 1 year

	Univariate analysis			Multivariate analysis		
Variables	HR	95% CI	p	HR	95% CI	p
Age	1.026	1.001–1.051	0.360			
Gender	0.884	0.642–1.216	0.448			
Ejection fraction	0.951	0.917–0.987	***0.007***	1.007	0.967–1.049	0.736
Hemoglobin	0.846	0.721–0.993	***0.041***			
Sodium	0.956	0.859–1.064	0.411			
BUN	1.024	1.017–1.032	***< 0.001***	1.017	1.008–1.027	***< 0.001***
Log BNP	2.074	1.664–2.585	***< 0.001***	1.695	1.347–2.134	***< 0.001***
Systolic blood pressure	0.972	0.958–0.986	***< 0.001***	0.982	0.969–0.995	***0.008***
High sensitive troponin	22.765	3.155–164.252	***0.002***	16.717	0.960–291.250	0.053

*BUN* blood urea nitrogen, *Log BNP* brain natriuretic peptide logarithmic

Variables which univariate analysis resulted in *p* < 0.04 were included in a multivariate analysis

We have also analyzed the composite endpoint of death and hospitalization in 1 year (Table 3). After multivariate adjustment, BUN (hazard ratio [HR] 1.008; 95% CI 1.001–1.015), Log BNP (hazard ratio [HR] 1.338; 95% CI 1.158–1.545), high sensitive troponin (hazard ratio [HR] 8.801; 95% CI 1.824–42.466) and age (hazard ratio [HR] 1.026; 95% CI 1.010–1.043) were associated with death and hospitalization within 1 year.

**Table 3:** Variables associated with hospitalization and all-cause mortality at 1 year

	Univariate analysis			Multivariate analysis		
Variables	HR	95% CI	p	HR	95% CI	p
Age	1.036	1.020–1.053	***< 0.001***	1.026	1.010–1.043	***0.001***
Gender	1.004	0.688–1.464	0.985			
Ejection fraction	0.975	0.954–0.996	***0.020***	0.991	0.967–1.015	0.443
Hemoglobin	0.848	0.767–0.936	***0.001***	0.917	0.825–1.019	0.107
Sodium	0.935	0.874–1.001	0.055			
BUN	1.017	1.011–1.022	***< 0.001***	1.008	1.001–1.015	***0.019***
Log BNP	1.547	1.359–1.761	***< 0.001***	1.338	1.158–1.545	***< 0.001***
Systolic blood pressure	0.991	0.983–0.999	***0.024***	0.994	0.986–1.002	0.129
High sensitive troponin	16.398	4.368–61.562	***< 0.001***	8.801	1.824–42.466	***0.007***

*BUN* blood urea nitrogen, *Log BNP* brain natriuretic peptide logarithmic

Variables which univariate analysis resulted in p < 0.04 were included in a multivariate analysis

**Fig. 2:**
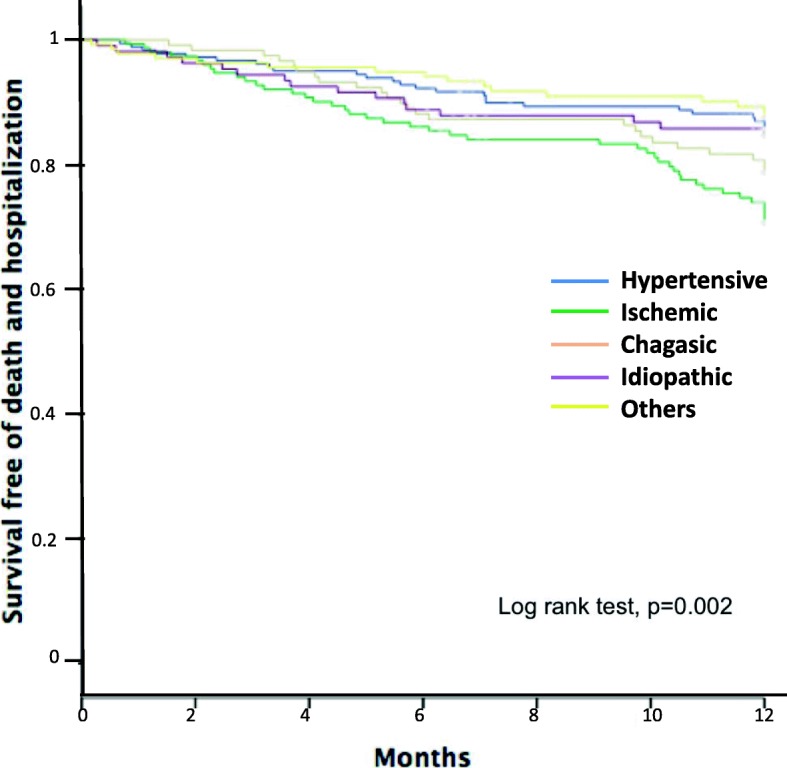
Death and hospitalization for all heart failure etiologies. Shows Kaplan Meier curves for all etiologies with death and hospitalization in 12 months

In Fig. 2, Kaplan Meier curves compared all etiologies. Ischemic patients had worse survival free of death and hospitalization followed by chagasic and idiopathic compared to other etiologies.

## Discussion

We observed an overall mortality of 6.8% and a composite outcome of death and hospitalization of 17.7% in 1-year of follow-up. It is known that the HF mortality increases with the follow-up time and can reach a median of 40% in 2.5 years [13]. Our results are in agreement with previous reports. For instance, Maggioni et al. observed all-cause mortality rate at 1 year of 7.2% in chronic stable HF in a pilot study [14]. In addition, the continuation of this study showed all-cause 1-year mortality rate of 6.4% and combined endpoint of mortality or HF hospitalization within 1 year of 14.5% [15].

In our cohort, the variables associated with all-cause mortality at 1 year were elevated BUN or log BNP and lower SBP. On the other hand, considering the composite endpoint death and hospitalization, the predictors were age, high sensitive troponin, BNP and BUN. Previous studies have shown a variety of risk predictors [13, 16, 17] and between then, it is common to find creatinine as representative of renal function and systolic blood pressure. However, although BNP is markedly related to prognosis [18, 19] it is not present in the best-known risk models [13, 16]. Due to the fact that studies in chronic heart failure are scarce in the Brazilian population, there are no known critical variables in our population, except in acute patients as in the BREATHE Registry [20].

The majority of included individuals were in NYHA class I/II (81%) denoting the stable-outpatient character population. Besides, the use of beta-blocker (96.8%) and ACEi or ARB (91%) was high. These facts refer to the good clinical care of the cohort, certainly influencing outcomes.

There was a predominance of hypertensive (26.0%) and ischemic (21.9%) etiologies in our sample, on the other hand, the number of chagasic patients was also important (17.0%). Most studies describe ischemic as the main etiology found in the HF population, however, it depends on study design and ascertainment approaches [5]. Nonetheless, in our population, Chagas disease is still a major concern. In Brazil, there are endemic areas of Chagas’ disease such as the Midwest region [21] and migration movements can explain this relative high prevalence. Recently, Nadruz et cols evaluated the population attributable risk (PAR) of Chagas cardiomyopathy for 2-year mortality among patients with HF enrolled at years 2002–2004 (era 1) and 2012–2014 (era 2). The era 2 population is part of our cohort and the results found were that although the absolute death rates decreased over time in the Chagas cardiomyopathy and non-Chagas cardiomyopathies groups, the PAR of Chagas cardiomyopathy for mortality increased among patients with HF. Therefore, the current knowledge indicate that of all etiologies, Chagasic HF has the worst prognosis [22]. In addition, Bernardez-Pereira et al. analyzed the association between genetic ancestry, self-declared race and hemodynamic parameters in the GENIUS-HF cohort and observed a predominance of European ancestry in the entire study population [23].Taken together, these facts make our cohort *sui generis* and suggest care in the use of risk prediction models from other populations.

The SEATTLE heart failure model was an example of a web-based calculator that estimates 1, 2 and 3-year survival using clinical and pharmacological data easily obtained, but could need calibration in different ethnic populations [16]. Regardless, many publications on HF risk scores in Europe, North America [13, 16, 17] and nowadays-in Asian populations [6–8], there is a shortage of studies in the Brazilian population.

We found that patients with ischemic etiology have worse survival free of death and hospitalization in 12 months compared to others etiologies. This is a common observation to many reports and also supports prior community-based epidemiological studies that reported greater risks of coronary heart disease-related deaths [24].

### Limitations

This study was specifically designed to study predictors of clinical deterioration in patients with reduced systolic fraction. For this reason we did not perform a subgroup analysis about preserved and mid range fraction patients.

This was a single-center study and, thus, our results might not be applicable to other populations. Nevertheless, the Heart Institute is a nation-wide referral center and our enrolled sample has individuals from different regions of the country.

## Conclusions

High levels of BUN and BNP and low systolic blood pressure were independent predictors of one-year overall mortality in our population. Considering the composite endpoint death and hospitalization, independent predictors were age, high sensitive troponin, BNP and BUN.

## Data Availability

The datasets analyzed during the current study are available from the corresponding author on reasonable request. They were collected and managed using REDCap electronic data capture tools hosted at the Clinical Hospital from University of São Paulo Medical School.

## Abbreviations

ACE: Angiotensin converting enzyme
ACEi: Angiotensin converting enzyme inhibitor
ARB: Angiotensin II receptor blocker
BMI: Body mass index
BNP: Brain natriuretic peptide
BUN: Blood urea nitrogen
CABG: Coronary artery bypass grafting
CKP-EPI: Chronic disease epidemiology collaboration
COPD: Chronic obstructive pulmonary disease
HF: Heart failure
HIV: Human immunodeficiency virus
LVDD: Left ventricular end diastolic dimension
NYHA: New York heart association
PCI: Percutaneous coronary intervention
SBP: Systolic blood pressure
